# Validation of a Deep Learning–Assisted Evaluation of Total Corneal Endothelial Cells Viability

**DOI:** 10.1167/tvst.14.9.20

**Published:** 2025-09-15

**Authors:** Matteo Airaldi, Filippo Airaldi, Zhuangzhi Gao, Alessandro Ruzza, Mohit Parekh, Diego Ponzin, Stephen Kaye, Francesco Semeraro, Stefano Ferrari, Yalin Zheng, Vito Romano

**Affiliations:** 1 Department of Molecular and Translational Medicine, University of Brescia, Brescia, Italy; 2 St Paul's Eye Unit, Liverpool University Hospitals NHS Foundation Trust, Liverpool, UK; 3 Department of Eye and Vision Sciences, Institute of Life Course and Medical Sciences, University of Liverpool, Liverpool, UK; 4 Delft Center for Systems and Control, Delft University of Technology, Delft, The Netherlands; 5 Fondazione Banca degli Occhi del Veneto, Venezia, Italy; 6 Schepens Eye Research Institute of Massachusetts Eye and Ear, Boston, MA, USA; 7 Ophthalmic Unit, ASST Spedali Civili di Brescia, Piazzale Spedali Civili, Brescia, Italia; 8 Department of Medical and Surgical Specialties, Radiological Sciences, and Public Health, University of Brescia, Brescia, Italy

**Keywords:** corneal transplantation, corneal endothelium, cell viability, graft survival

## Abstract

**Purpose:**

To describe the validation of a novel automated analysis of preoperative pan-corneal endothelial cell viability.

**Methods:**

Preclinical experimental study. Dead endothelial cells and denuded areas of Descemet membrane of corneoscleral rims were stained with trypan blue (TB) 0.05%. Endothelial mortality was estimated by an experienced eye bank technician (“gold standard”) and by deep learning–aided automated segmentation of TB-positive areas (TBPAs) on images of the stained corneas (“V-CHECK method”). V-CHECK mortality was calculated for the whole cornea and for concentric 2-mm rings. The agreement in the estimation of endothelial mortality between the two methods was assessed with Bland–Altman analysis and correlation tests.

**Results:**

Nineteen corneas deemed unsuitable for transplantation were used for the experiment. The automated V-CHECK method was able to accurately segment the corneal endothelium and the TBPAs. The gold standard and the V-CHECK method showed a strong positive correlation for all rings (Pearson's ρ, range 0.76–0.81, all *P* < 0.001). The V-CHECK method resulted in a higher average estimated endothelial mortality (mean difference range +6.5% to +9.5%).

**Conclusions:**

The V-CHECK method enables reproducible estimation of endothelial cell viability in donor corneas. Incorporating this technique into the preoperative assessment of donor corneal tissues (in the eye bank and in the operating theater) can provide a reliable evaluation of endothelial health, thereby improving the consistency of tissue quality and further supporting optimal surgical results.

**Translational Relevance:**

The V-CHECK deep learning–assisted computer vision protocol will allow surgeons and eye bank technicians to perform an independent, preoperative assessment of global corneal endothelial viability.

## Introduction

Quality control of donor tissue destined for keratoplasty is of utmost importance to ensure the safety and efficacy of corneal transplantation.^1^ Endothelial health status, in particular, is a major determinant of the long-term success of keratoplasty.^2^ Higher endothelial cell density (ECD) values have been associated with lower rates of long-term graft failure.^3^ Nevertheless, ECD only estimates the cellular density of selected areas of the endothelium and thus cannot adequately represent the full picture of corneal endothelial health.^4^^,^^5^

Although numerous preoperative assessments and graft selection criteria are employed to ensure a successful outcome prior to any kind of transplantation,^6^ corneal surgeons usually do not have any means to directly measure the endothelial cell density or viability of a donor graft in the operating theater, even if the responsibility for determining whether a donor cornea is suitable for transplantation ultimately rests with them.^1^

The development of a reliable and reproducible assessment of global endothelial health status of donor corneas would represent a powerful tool for corneal surgeons, and it might not only contribute to the exclusion of poor-quality tissues but also lead to a greater standardization of keratoplasty outcomes.^1^^,^^7^

In this article, we validate an automated software for the preoperative evaluation of the corneal endothelial cell viability that can be used to obtain reliable descriptors of global corneal endothelial health. This software can be used for further analysis of large image data sets to correlate preoperative evaluated endothelial mortality with well-established parameters of corneal tissue quality, such as endothelial cell density and eye bank–declared endothelial mortality.

The protocol described in this article will be used for the image analysis of the Viability Control of Human Endothelial Cells before Keratoplasty longitudinal study (V-CHECK Study, NCT05847387),^7^ a cohort study that aims to correlate the preoperative evaluation of endothelial viability with 1-year clinical outcomes of patients undergoing keratoplasty.

## Materials and Methods

This preclinical experimental study for the validation of a new corneal endothelial cell viability assessment was conducted at the Veneto Eye Bank Foundation, Venice, Italy. Donor corneal tissues unsuitable for transplantation were used for the research purposes and the validation studies described in this article, according to the provisions of the Italian law 91/99 and after an informed consent form was signed by the donor's next of kin. The study was conducted in accordance with the tenets of the Declaration of Helsinki. This study has been evaluated by the institutional review board of the Veneto Eye Bank Foundation and deemed not to require ethics approval. The human tissue experiments complied with the guidelines of the ARVO Best Practices for Using Human Eye Tissue in Research.

### Live/Dead Staining

Exploratory live/dead assays aimed to verify an acceptable correlation between live/dead staining with trypan blue and calcein acetoxymethyl (Calcein AM) to confirm the validity of the V-CHECK endothelial evaluation protocol.

**Figure 1.**
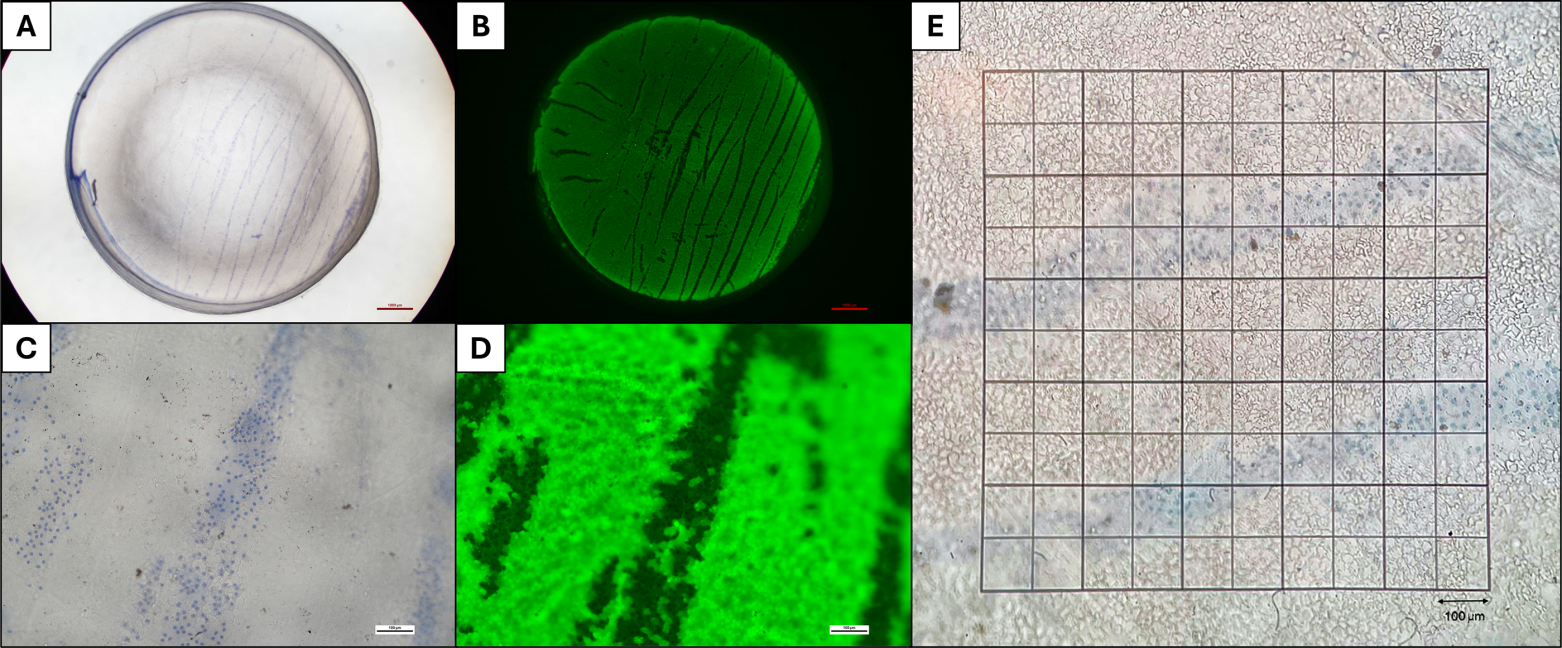
Live/dead analysis of the whole cornea. (**A**) Staining with TB 0.05% for 45 seconds highlights the presence of TBPA. (**B**) After incubating the whole cornea with Calcein AM at 37°C for 20 minutes, the same linear areas of endothelial damage are visible. (**C**, **D**) High-magnification images of **A** and **B** highlight the near-perfect inverse correlation between areas of endothelial cell death and of denuded Descemet membrane in **C** with metabolically active endothelial cells in **D**. (**E**) The reticule used for manual counting of TBPA (i.e., gold standard method). The 10 × 10 mm reticule, observed with an inverted stereoscopic microscope with a 10× magnification, results in a 1-mm^2^ overall area subdivided in 100 smaller squares, each with a 10^4^-µm^2^ area. The percentage of TBPA in the gold standard method was estimated by counting the total surface of stained areas over the total area of the endothelium.

Trypan blue is a vital dye commonly used in anterior segment surgery, which stains the nuclei of severely damaged and dead endothelial cells of donor corneas, as well as areas of Descemet membrane denuded of endothelial cells.^8^ Calcein AM is a hydrophobic compound that permeates intact live cells. After incubation at 37°C for 20 minutes, hydrolysis by ubiquitous intracellular esterases produces a green fluorescent product. Figures 1A–D show areas of mortality from a whole cornea stained with 0.05% trypan blue (TB) for 45 seconds, demonstrating near-perfect correlation with Calcein AM staining of the same tissue.

### Gold Standard: Manual Endothelial Viability Assessment

A previously described light microscopy assessment was used as the gold standard for the estimation of endothelial mortality of included corneas by manually counting the percentage of TB-positive area (TBPA) over the entirety of the endothelium.^9^ Once removed from their storage vessel, corneal tissues deemed unsuitable for corneal transplantation were thoroughly rinsed in balanced salt solution (BSS) to remove excess transport medium. The endothelium was then stained for 30 to 45 seconds with TB 0.05%. A 10 × 10 mm reticule was inserted into an inverted stereoscopic microscope with 10× magnification, resulting in a 1-mm^2^ overall area subdivided into 100 smaller squares, each with a 10^4^-µm^2^ area, as shown in Figure 1E. The percentage of TBPA was estimated by counting the total surface of stained areas over the total area of the endothelium. Values of TBPA obtained with this method by an experienced eye bank technician (AR) served as the “gold standard" for the comparison with the V-CHECK method.

### V-CHECK—Image Acquisition

After manual counting, the corneal tissues were positioned under a surgical microscope for the acquisition of images suitable for the V-CHECK analysis. The corneal tissue was placed face-up on a base without suction holes to avoid background optical disturbances that could prevent optimal image analysis of the central endothelium. The corneoscleral rim was filled with BSS to avoid the formation of reflexes of the microscope lights on the central endothelium. A short visual explanation of the procedure is available in the eye bank setting in Supplementary Video S1, highlighting its use for tissue inspection before tissue preparation, and in an operating theater setting in Supplementary Video S2, before the implantation of a precut ultrathin Descemet stripping automated endothelial keratoplasty (UT-DSAEK) graft. To acquire images suitable for analysis, the magnification level of the surgical microscope must be adjusted to make sure that the entirety of the Schwalbe line is visible just inside the image frame. Images of the stained corneal endothelium are then acquired and exported from the surgical microscope.

### V-CHECK—Image Analysis

Exported images were then analyzed with the V-CHECK method. The analysis consists of three sequential steps: (1) the segmentation of corneal tissue from each surgical microscope image, (2) the segmentation of TBPAs in each corneal image, and (3) the computation of its estimated viability. The computer vision algorithm described below was implemented and carried out in Python 3.12 with OpenCV.^10^

#### Segmentation of Corneal Endothelium via the Segment Anything Model

The goal of the first step is to segment corneas from the original surgical microscope images, that is, separate pixels belonging to the corneal endothelium from background pixels (e.g., trephine base, surgical tray).

To enable this segmentation in an automatic way, we adopted the Segment Anything Model (SAM).^11^ SAM is a foundation model, trained on a large quantity of data, which has shown strong zero-shot generalization capabilities (i.e., handling tasks or making predictions for cases it was not explicitly trained for), sparking significant interest in its application to a wide range of tasks, including biomedical segmentation.^12^^,^^13^ SAM is also categorized as a promptable segmentation model (i.e., the user can provide, alongside the input image, one or more prompts specifying what needs to be segmented). Such prompts can be either sparse (text, bounding boxes, points of interest) or dense (masks).

**Figure 2.**
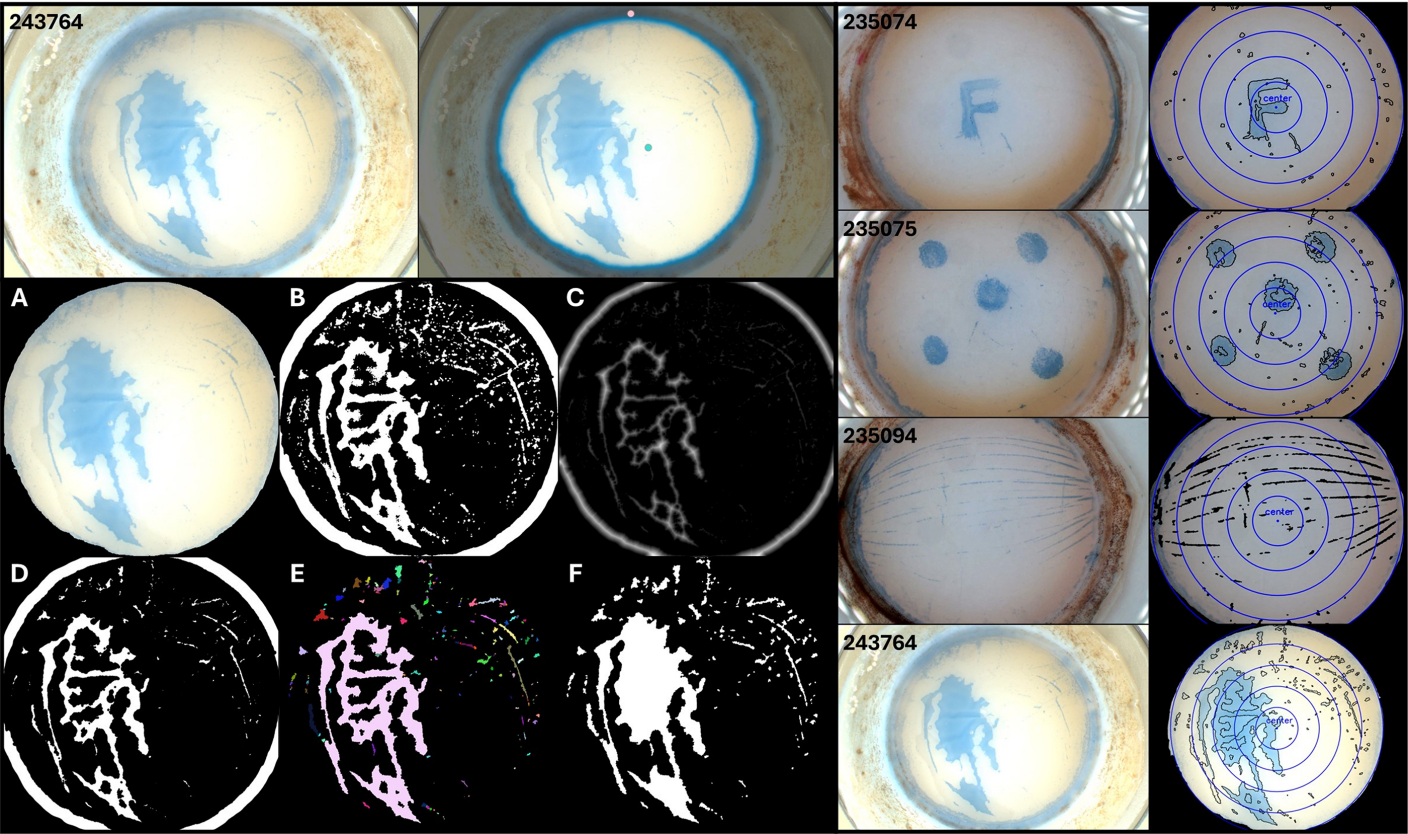
*Left* *,* *top*: example of segmentation of the corneal endothelium from a microscopic image via SAM. On the *top left*, the input image is shown. On the *right*, the user specified two points of interest: in green, the region to be segmented; in pink, regions to be excluded from the segmentation. *Left, bottom*: stages of the TBPA segmentation algorithm. (**A**) The final output of SAM endothelial segmentation (i.e., the input image). (**B**) The filtered and contrast-adjusted grayscale conversion. (**C**) The distance transform. (**D**) The thresholded foreground, indicating likely TB-stained regions. (**E**) The output of the watershed algorithm, where each TBPA is assigned a random color. (**F**) Contouring and conversion of the watershed output as a mask of the TBPAs. *Right*: examples of input images and processed images. *Left column*: surgical microscope view of donor corneal tissues after 45 seconds of staining with 0.05% TB, showing areas of endothelial damage in different configurations, some of which were mechanically induced (top two images). Areas of endothelial damage are readily identified by TB staining, indicating the presence of areas of endothelial cell death or denuded Descemet membrane. *Right column*: after deep learning segmentation of the corneal surface and automated isolation of the stained areas, the TBPA can be reliably quantified.

In our case, SAM was consistently able to segment the corneal endothelium from the rest of the image when prompted with one or more points. The scleral rim and the image background detected by SAM were automatically removed from the images, thus yielding the endothelium image ready for further analysis in the next step. Figure 2 (left, top) showcases an example of the segmentation process.

#### Segmentation of TBPAs

Once the corneal endothelium surface has been isolated, the TBPAs can be segmented against the remaining regions of nonstained, healthy tissues via an automated computer vision algorithm. The algorithm consists of the following operations:

1. The input image is converted from RGB space to grayscale, and a Gaussian blur is applied to reduce noise and to smoothen intensity variations (Fig. 2A). For images with insufficient contrast, contrast-limited adaptive histogram equalization (CLAHE) is performed before the Gaussian filter in order to locally enhance contrast.^14^
2. Adaptive thresholding is applied to the filtered gray image to coarsely identify potential TBPAs. This technique leverages variations in the intensity of pixels in local neighborhoods to generate a preliminary mask highlighting candidate stained regions. To enhance segmentation quality, small nonzero artifacts in the image are removed after thresholding via morphological opening, which helps isolate larger contiguous regions while removing smaller noise (Fig. 2B).
3. A distance transform is computed for each nonzero pixel in the mask, transforming pixel values to represent their distance to the nearest background pixel (Fig. 2C).
4. A second threshold is applied to the distance-transformed image, creating a binary map that distinguishes likely foreground regions (high-intensity areas, representing proximity to TB-stained regions) from background and unknown regions, likely nonstained healthy area (Fig. 2D).
5. The segmented foreground is used as a seed in a watershed algorithm.^15^ Prior to watershed flooding, pixels outside the cornea are uniformly labeled to mitigate edge artifacts (otherwise, the boundary of the cornea could disrupt the segmentation), and unknown regions are grouped as a single region, enhancing segmentation precision. The watershed algorithm then iteratively refines region boundaries, resulting in well-defined segments for each candidate TB-stained area (Fig. 2E).
6. Finally, contours of the regions segmented by the watershed algorithm are extracted by thresholding and filtering to retain only those within the corneal mask. These contours are drawn over a mask that serves as the final TBPA indicator, highlighting areas stained by TB within the corneal region (Fig. 2F).

#### Viability Assessment

In the last step, given the segmented TBPAs, the V-CHECK viability assessment can be performed. To improve its interpretability, we divided the corneal endothelium segmented by SAM into five concentric rings, with radii approximately at 2, 4, 6, 8, and 10 mm from the centroid of the image. This centroid is extracted from the image moments. The radius of the outermost ring is computed in a least squares manner as the average of the distances between the centroid and each point on the contour of the cornea. The other radii (i.e., at 2, 4, 6, 8 mm) are subsequently found as the corresponding fraction of the outermost radius. Given that the outer ring's radius is computed via least squares and that the cornea is not a perfectly circular objective, a mismatch between the outer ring and the whole cornea is possible. For this reason, subsequent analyses report the outcomes for both the 10-mm ring and the whole cornea.

For each ring, the percentage of TBPAs is calculated as the ratio of the number of positively segmented pixels lying within the disc, over the total count of corneal pixels in the area.

### V-CHECK—Validation

To investigate the reproducibility of the proposed V-CHECK method analysis, all available images were rotated by 90°, 180°, and 270° and then segmented and analyzed again with the aforementioned methodology. The goal was to verify the repeatability of the TBPA estimation, as well as to test its invariance and robustness with respect to rotations of the corneal images.

To further confirm the accuracy of the V-CHECK segmentation, a comparison with a manual ground truth was performed. In particular, the central, 2-mm ring of each image was segmented with the SAM tool. The image was then analyzed (1) with the V-CHECK method and (2) via a semiautomated approach by loading the segmented image into ImageJ (National Institutes of Health, Bethesda, MD, USA). The loaded image was converted into an RGB stack, and the green channel was selected for further processing. CLAHE was then performed in order to optimize the contrast (with a block size of 127 pixels, 256 histogram bins, maximum slope of 3), before obtaining a binarized mask through thresholding. The threshold value was manually adjusted for each image until the segmentation of TBPA was deemed adequate.

### Statistical Analysis

Descriptive data were summarized using mean (standard deviation [SD]), median (interquartile range [IQR]), and 95% confidence intervals (CIs) where appropriate.

The distribution of V-CHECK–measured mortality for each of the 0°, 90°, 180°, and 270° rotations (with 0° being the original image) and for each of the ring segmentations was converted to *z*-scores to allow for comparison of the different tissues on the same normalized scale. The distribution of V-CHECK mortality *z*-scores was displayed by means of violin plots and boxplots, and Levene's tests were used to verify homoscedasticity (i.e., equal variance) of the values across the different rotations for each ring.

The agreement between semiautomated segmentation of TBPA areas and V-CHECK segmentation was then evaluated by comparing the two binarized images of the central ring of each cornea. Various metrics, commonly employed in segmentation tasks, were calculated for each image pair and their distribution displayed via boxplots. These included precision and recall of TBPA areas, the balanced accuracy (i.e., the average of the recall of stained and nonstained areas), the Dice coefficient and intersection over union (also known as F1 score and Jaccard index, respectively), and the standard and modified Hausdorff distances (normalized by the size of each image).^16^ Continuous performance metrics were computed to assess the agreement in predicted TBPA extent, including mean absolute error (MAE), root mean squared error (RMSE), and the coefficient of determination (*R*²). Bootstrapping with 1000 resamples was used to estimate 95% CIs for MAE, RMSE, and *R*².

Pearson's correlation coefficients were calculated to quantify the correlation between mortality as measured by an expert eye bank technician (gold standard) and mortality measured with the V-CHECK method. Scatterplots were employed to visualize the correlation between the two modalities.

Bland–Altman plots were used to assess mean differences and the 95% limits of agreement between the two techniques.

All statistical analyses were conducted using R software version 4.2.2 (R Project for Statistical Computing, Vienna, Austria).

## Results

A total of 19 corneas deemed unsuitable for transplantation and destined for research purposes were included in the study.

**Figure 3.**
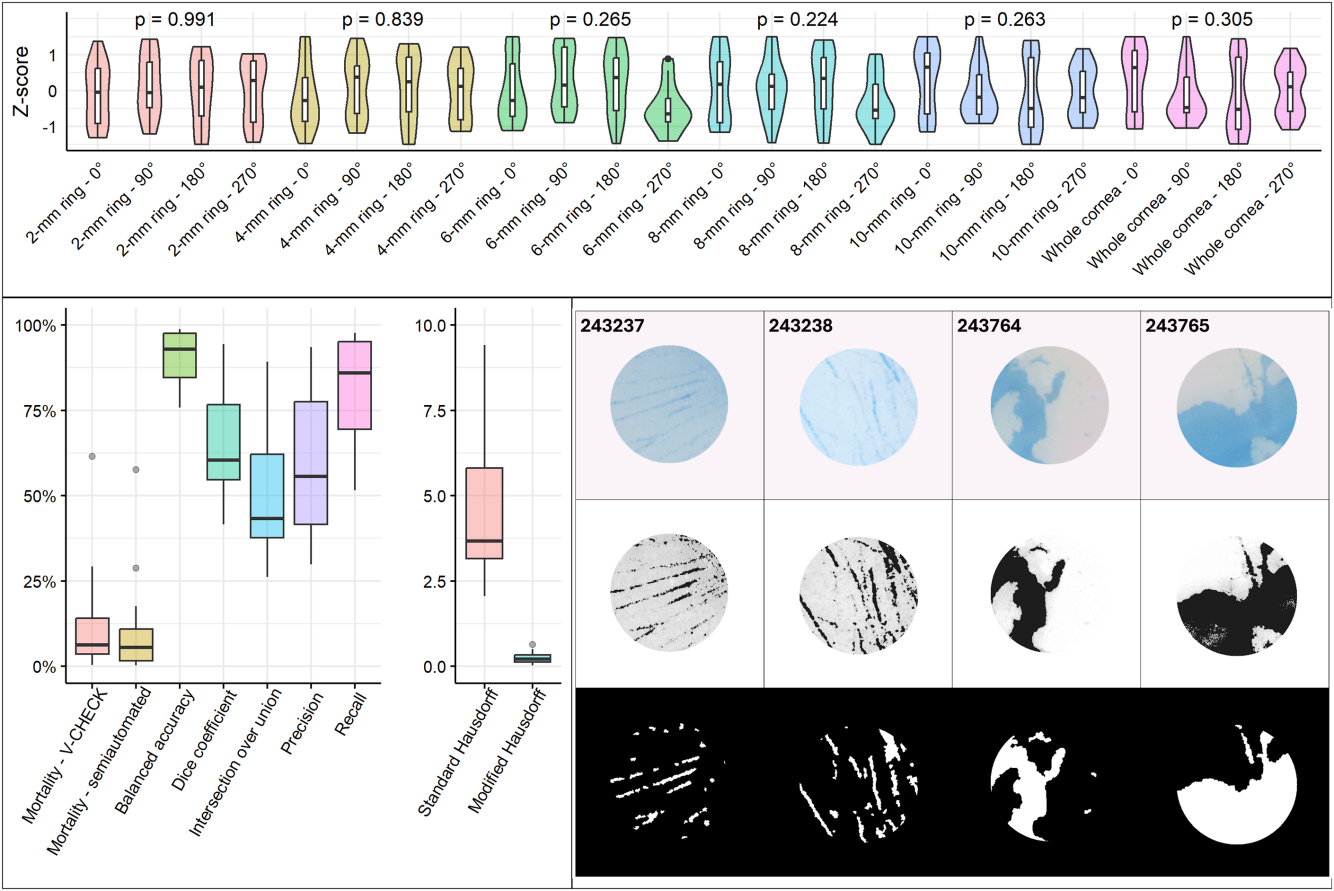
*Top*: violin plots and boxplots displaying the distribution of *z*-scores of endothelial mortality as measured by the V-CHECK method across all rings of the image for 0°, 90°, 180°, and 270° rotations. Levene's tests demonstrated homogeneous variance of mortality for all rings and rotations, reflecting the repeatability of the V-CHECK method. *Bottom, left*: boxplots displaying metrics of concordance between the semiautomated segmentation of TBPAs (ground truth) and the V-CHECK method, which performed with high balanced accuracy and good Dice coefficient, reflecting the overlap of the two segmented masks. Hausdorff distance scales are in millimeters. *Bottom, right*: examples of central 2-mm segmented corneas (*top strip*), the ground truth, i.e. semiautomated thresholding performed with ImageJ (*middle strip*), and the V-CHECK method segmentation (*bottom strip*).

The estimation of endothelial mortality with the V-CHECK method demonstrated good reproducibility, with constant variance across different rotations of the same images. As shown in Figure 3 (top), *z*-scores of V-CHECK mortality in each ring had homogeneous variance across the different rotations (Levene's tests, all *P* > 0.05).

The V-CHECK method also demonstrated good overall agreement with the ground truth from semiautomatically segmented images of the central 2-mm corneal ring. Quantitative metrics comparing the two segmentations, along with representative binary masks, are shown in Figure 3 (bottom). V-CHECK achieved high balanced accuracy (median [IQR]: 91.4% [84.4%, 96.6%]) and a good Dice coefficient (60.3% [54.3%, 75.6%]), reflecting substantial overlap between predicted and reference TBPA regions. The corresponding intersection over union (IoU) was 43.3% (37.7%, 62.1%). The method showed strong recall (85.9% [69.4%, 95.1%]) with moderate precision (55.6% [41.6%, 77.5%]), suggesting slight oversegmentation. Spatial accuracy was supported by a low modified Hausdorff distance (median: 0.20 mm [0.11, 0.35]). In terms of continuous prediction accuracy, the model achieved an MAE of 0.019 (95% CI, 0.012–0.026), an RMSE of 0.024 (95% CI, 0.016–0.030), and an *R*^2^ of 97.4% (95% CI, 67.7%–98.9%) when comparing predicted and ground-truth mortality fractions. The average (SD) mortality within the 2-mm ring predicted by the V-CHECK was slightly higher than the ground truth (11.8% [15.7%] vs. 10.4% [15.2%]), suggesting a modest positive bias in the segmentation output.

The overall, pan-corneal endothelial mortality of the 19 tissues according to the V-CHECK method and that measured through manual counting under light microscopy by an experienced eye bank technician (i.e., the gold standard) are reported in the Table. Example images of tissues analyzed with the V-CHECK method are displayed in Figure 2 and Figure 3.

**Table.** Average Mortality Values and Differences Between the V-CHECK Method and the Gold Standard (Manual Counting) Method

Method	Average Mortality, Mean (SD)	Average Difference GS vs. V-CHECK, Mean (95% LoA)
Gold standard		
Whole cornea	9.1% (17.7%)	
V-CHECK		
Whole cornea	15.9% (11%)	6.8% (−14.7%, 28.2%)
Ring 1	18.7% (21.4%)	9.5% (−17.8%, 36.9%)
Ring 2	15.7% (18.7%)	6.6% (−18.1%, 31.4%)
Ring 3	15.6% (15.9%)	6.5% (−16.8%, 29.8%)
Ring 4	16.7% (13%)	7.6% (−13.5%, 28.6%)
Ring 5	16.1% (11.1%)	7% (−14.5%, 28.5%)

GS, gold standard; LoA, limits of agreement.

**Figure 4.**
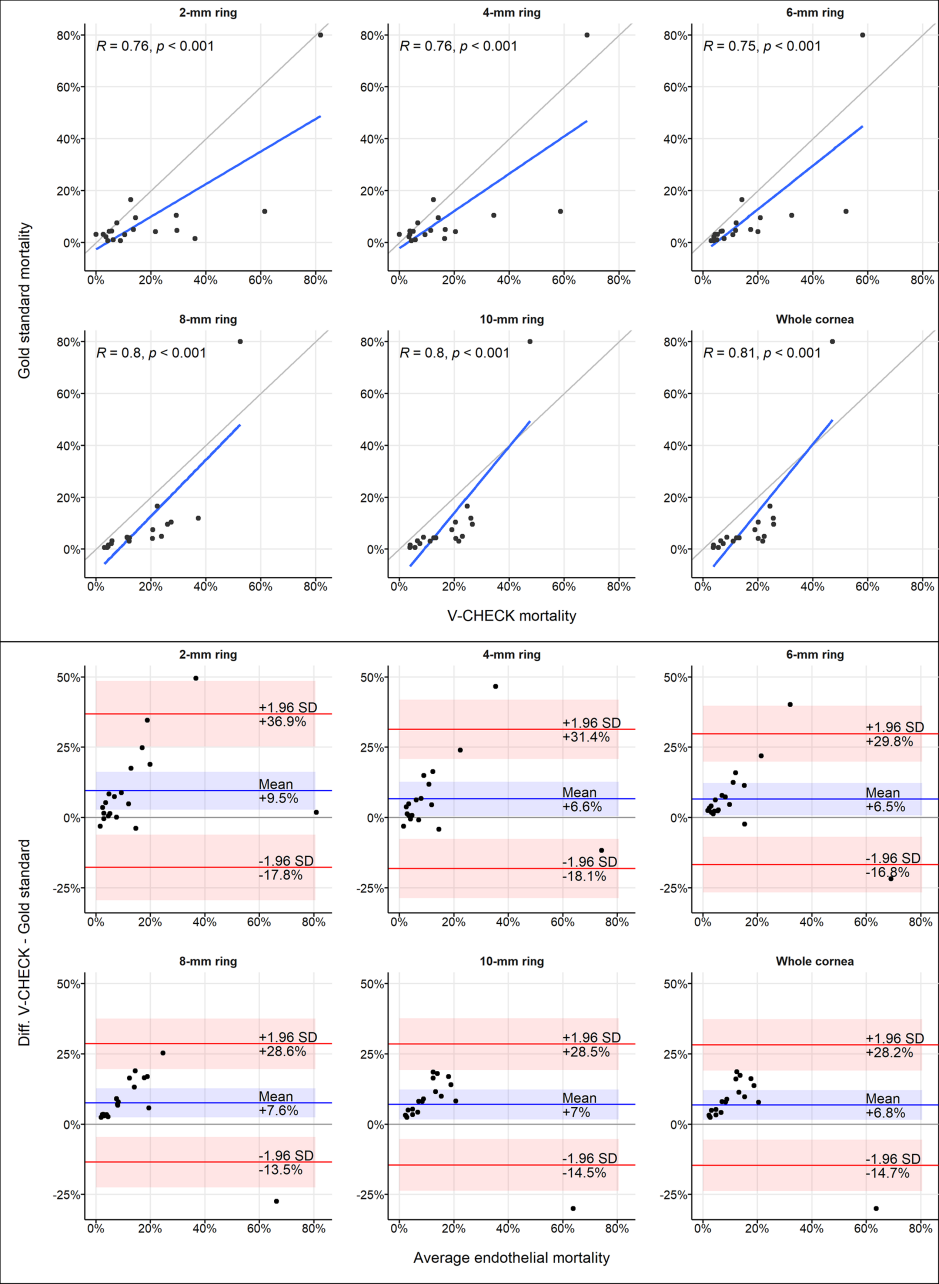
*Top*: scatterplots showing the correlation between gold standard mortality and V-CHECK mortality. The two methods showed strong positive correlation in all rings (Pearson's ρ, range 0.76–0.81, all *P* < 0.001). *Bottom*: Bland–Altman analysis of the agreement between measurements of endothelial mortality from the gold standard method (i.e., manual counting of TB-positive cells) and the V-CHECK method. The V-CHECK method produced higher estimates of endothelial mortality than manual counting (mean difference range [+6.5% to +9.5%]). The *blue line* represents the mean of the differences between the two methods, while the *red lines* represent the 95% limits of agreement. The *shaded ribbons* represent the respective 95% CI.

The gold standard and the V-CHECK method showed a strong positive correlation of mortality estimates in all rings (Pearson's ρ, range 0.76–0.81, all *P* < 0.001). Scatterplots displaying the positive correlation of V-CHECK mortality and gold standard mortality assessments are displayed in Figure 4 (top).

The gold standard and V-CHECK method also showed an overall acceptable agreement in the measurement of pan-corneal endothelial viability. The V-CHECK method resulted in a higher average estimated endothelial mortality, with the average difference in TBPA estimation ranging from +6.5% to +9.5% across the different rings (Table). The results of the Bland–Altman analysis for each of the concentric rings, reporting the average difference between the estimated mortality by the two methods and the respective 95% limits of agreement, are displayed in Figure 4 (bottom).

## Discussion

In this study, we validated an automated software tool for assessing corneal endothelial cell viability in donor corneal tissues. The V-CHECK method provides eye bank technicians with an objective, comprehensive assessment of endothelial viability, which is essential for determining tissue suitability. Additionally, the V-CHECK method enables corneal surgeons to perform a final evaluation of endothelial health directly in the operating room prior to corneal transplantation. By providing a comprehensive, real-time assessment of endothelial health status, the V-CHECK method offers a valuable and robust quality assurance measure for donor tissues, supporting more informed surgical decision-making and potentially enhancing patient outcomes in corneal transplantation.

Endothelial cell density (ECD) currently represents the single most important indicator of donor corneal tissue status, and its relevance as a determinant of long-term graft survival has long been proven.^3^ The minimum requirements for delivering a donor cornea might vary from eye bank to eye bank, but ECD is universally adopted to set a threshold for transplantation suitability. This metric is one of the few indicators of endothelial status available to the surgical team performing the keratoplasty, yet ECD does not necessarily reflect total corneal endothelial health.^17^^,^^18^ Pipparelli et al.^4^ compared ECD to viability assays and found that the actual number of living, functional endothelial cells of donor corneas was significantly lower than the ECD reported by standard eye bank methods, with a median reduction of approximately 12% to 20% depending on the preparation and handling of the tissue. This discrepancy might explain the rapid early postoperative decline in ECD observed after keratoplasty, suggesting that fewer viable cells are transplanted than the preoperative ECD would indicate. Large-scale analyses of eye bank data have also revealed systematic anomalies in ECD reporting. An analysis of 48,207 eye bank reports by Munir and Munir^19^ revealed a discontinuity in reported ECD values just below commonly used clinical thresholds (e.g., 2500 cells/mm²), with a clustering of values at or just above these cutoffs. This pattern is possibly explained by subjective human bias during the counting process, where supra-threshold values might be more likely to be reported to meet suitability criteria for transplantation. This further supports the claim that ECD, as measured in the eye bank setting, may be biased toward overestimating tissue viability. Given the inherent limitations of ECD, most eye banks, especially in Europe, also directly evaluate endothelial cell viability, specifying a 5% mortality threshold to be deemed suitable for transplantation.^20^

There is a need for a greater degree of standardization of outcomes, both functional and survival-wise, in corneal transplant surgery, a demand whose satisfaction is strongly dependent on developing metrics that better reflect the global endothelial health of donor tissues.^21^ Pan-corneal assessments of endothelial viability in donor tissues have highlighted how ECD probably overestimates true cell density,^4^^,^^5^ but these methods, even though they allow a global evaluation of endothelial status, are not feasible in the theater as they are destructive to the graft.^4^^,^^5^^,^^22^^–^^26^ With the possibility of a final endothelial health assessment in the surgical setting before transplantation, the V-CHECK method will instead allow corneal surgeons to estimate the true final endothelial viability of the graft. In fact, endothelial cell loss (ECL) generated during the storage time between graft preparation in the eye bank and corneal transplantation, which can induce mortality, especially if preserved for a longer period of time, is usually not accounted for.^22^ This technique could potentially highlight true surgical outcomes since the ECL just prior to surgery will be accounted for. The V-CHECK method therefore offers a reliable and comparable viability analysis, accounting for ECL just prior to transplantation and helping to determine the surgically induced ECL after the corneal graft is transplanted.

The feasibility of our method in the preoperative setting is not its only advantage. Compared to pan-corneal evaluation with the triple staining method, employing a Hoechst/ethidium-homodimer/Calcein AM combination,^4^^,^^22^ the simpler pan-corneal live/dead analysis that we described does not present the risk of overestimating the true damage to donor corneal tissues, as only dead cells or denuded areas of Descemet membrane get stained by trypan blue. For instance, it has previously been noted that under triple-staining coloration, preloaded Descemet membrane endothelial keratoplasty (DMEK) tissues exhibit high levels of cells that are not actively converting the Calcein AM to calcein, but whose membranes are not yet permeable to ethidium, meaning they have been damaged but are not yet apoptotic.^27^ This population of cells will not be represented as TBPA in the V-CHECK method, guaranteeing that no overestimation of the true endothelial damage occurs and that donor tissues that might have been still employed for transplantation are excluded.

With regard to safety concerns, it is recognized that higher concentrations and prolonged exposure times to TB are cytotoxic to corneal endothelial cells. In vitro and ex vivo studies demonstrate that TB concentrations commonly used in ophthalmic surgery (e.g., 0.06%–0.15%) and exposure times of 1 to 5 minutes do not result in significant corneal ECL or functional impairment and are considered safe for clinical use.^8^ Cytotoxicity becomes evident at higher concentrations (as low as ≥0.1%) and/or extended exposure (as low as ≥30 minutes) in an inversely proportional fashion, leading to significant endothelial cell loss and morphological changes.^8^^,^^28^ Short-term exposures (≤5 minutes) to 0.06% to 0.15% TB do not impair mitochondrial function or reduce ECD.^28^^,^^29^ The V-CHECK protocol employs staining with 0.05% TB for 30 to 45 seconds, well below the cytotoxic thresholds.^8^ In addition, 0.25% TB is commonly used by eye banks during multiple stages (stripping, evaluation, and loading) of membrane preparation for DMEK surgery, and no toxicity issues have been reported during validation studies.^30^ Of note, impurities in TB preparations have been associated with toxic anterior segment syndrome and complete ECL^31^; therefore, high-purity commercial preparations are recommended.

It might seem contradictory that in our validation experiments, the V-CHECK method produced higher estimated values of TBPAs compared to the gold standard (i.e., manual counting of TB-positive areas). The difference was relevant, with a range in average estimated mortality that was 6.5% to 9.5% higher in the V-CHECK method. However, the elevated correspondence in the inverted staining patterns of Calcein AM and TB, together with the strong agreement between the semi-automated, ImageJ-aided segmentation and the V-CHECK method, as well as the strong positive correlation of mortality scores between the V-CHECK and manual counting methods presented in this work, are all indicators of a high reliability of the V-CHECK method in assessing endothelial mortality. A crucial difference between the gold standard employed in this study and the V-CHECK method is that the latter aims to provide corneal surgeons with an overall evaluation of the global health of the endothelium, while manual counting in the eye bank is usually performed over a section of the central cornea (1 mm^2^) such as in the present work.^9^ This discrepancy in the analyzed area might explain the difference in measurements between the two methods. It can be argued that the evaluation of a larger area of endothelium is more relevant to surgical outcomes of corneal transplant than that of only a central square of tissue. The V-CHECK method can be easily applied to the whole surface of the corneal endothelium, or it can be limited to a certain area of endothelium, for example, the 8-mm circle of a DMEK graft.

An approach similar to the V-CHECK method has been very recently proposed by Szkarlat et al.,^32^ who developed a TB-based, pan-corneal viability assessment after processing preloaded DMEK tissues in an eye bank setting. Interestingly, they found that visual assessment by experienced eye bank technicians produced similar estimations of global endothelial cell mortality compared to automated image analysis, while specular microscopy overestimated the ECD after DMEK preparation, again suggesting that ECD represents an inaccurate and poorly reproducible measure of endothelial viability. It is also important to note that estimation of endothelial mortality through automated image processing in Szkarlat et al.,^32^ as well as in other similar works analyzing DMEK tissues after preparation, frequently exceeds mortality values of 15% to 20%, underlying how the evaluation of the whole endothelium might frequently yield larger estimates of endothelial damage compared to ECD or visual inspection.^25^^,^^26^^,^^32^

Finally, in a recent article, Yoon et al.^33^ presented a comparable method to evaluate the damage to corneal endothelium after preparation of corneal tissues for transplantation. In their analysis, images of donor corneas stained with 0.06% TB were processed in an image analysis software using manual selection of stained areas. Compared to this technique, the V-CHECK method presents several advantages. First, the use of an automated, deep learning–based segmentation can allow the V-CHECK method to be applied to large data sets of images and to be implemented in the operating theater for real-time evaluation of endothelial damage.^7^ Furthermore, the use of a trephine base without suction holes avoids background disturbances that can affect the estimation of TBPAs. Likewise, the use of a BSS fill of the donor corneoscleral rim described in our image acquisition protocol reduces the glare and light reflexes from the surgical microscope, evident in the images from Yoon et al.,^33^ which again can affect the estimation of TBPAs.

A limitation of our current V-CHECK method is that we have not fine-tuned SAM (i.e., we employed the available pretrained model without adjustments to its weights). Despite the fact that SAM offers an adequate and reliable off-the-shelf tool, we hypothesize that additional training on even a small curated data set of microscopy images via state-of-the-art fine-tuning methodologies, such as Conv-LoRA,^34^ could yield improved segmentation results. This would also have the benefit of streamlining and automating the process further, requiring fewer or no human prompt to detect corneal regions. Future developments of the V-CHECK method will therefore include these SAM optimizations. A further limitation of this study is the somewhat small sample size that might underrepresent the variability in mortality patterns, viability thresholds, or imaging strategies. However, given the high reproducibility demonstrated by the V-CHECK method in the present work, we are confident in the generalizability of its results. Nevertheless, with the collection of a larger data set through the longitudinal V-CHECK cohort study, we may be able to implement more robust and better-tested iterations of the V-CHECK tool that are less susceptible to biases in the pretrained, possibly fine-tuned model and misrepresentations in the corneal images.

In conclusion, the V-CHECK method provides an innovative and reliable approach for evaluating endothelial cell viability in donor corneas through automated, deep learning–based segmentation of TBPAs. This method demonstrated strong correlation with manual counting by experienced eye bank technicians, establishing its validity as a robust alternative for preoperative assessment. Unlike traditional methods, V-CHECK allows analysis of endothelial viability across the entire cornea—not limited to small, centralized samples of endothelium—providing eye bank technicians with a comprehensive tool for objectively reporting global endothelial health and enhancing quality assurance in donor tissue evaluation. Furthermore, the V-CHECK analysis enables corneal surgeons to directly assess endothelial status in the operating theater—a critical capability not currently available to them, despite surgeons bearing sole responsibility for transplantation success.^1^^,^^21^ By incorporating the V-CHECK method into their preoperative routine, surgeons may be better equipped to identify any issues with the donor corneal tissue that could impact graft survival, evaluate their technique, and compare with peers.

It is crucial to have accessible and reproducible methods for assessing endothelial status, as endothelial cell viability is one of the key factors in determining graft success and long-term outcomes. Prospective, large-scale studies are warranted to further validate the V-CHECK method and its role in enhancing donor corneal tissue quality assessments. In the ongoing V-CHECK study, a cohort of keratoplasty patients will be followed for 12 months to evaluate the correlation between preoperative endothelial viability, as measured by the V-CHECK method, and clinical outcomes.^7^ Integrating the V-CHECK analysis into routine preoperative evaluations in eye banks and operating theaters offers significant potential to standardize assessment protocols, optimize graft survival, and ultimately enhance patient outcomes in corneal transplantation.

## Supplementary Material

Supplement 1

Supplement 2

## Supplementary Material

Supplementary Video S1. V-CHECK in the eye bank setting. The V-CHECK can be easily employed in the eye bank for a further quality assessment step. In the video, a full donor corneoscleral rim is stained to evaluate its suitability for the preparation of a preloaded DMEK graft.

Supplementary Video S2. V-CHECK in the operating room. In the video, a precut ultrathin Descemet stripping automated endothelial keratoplasty graft (UT-DSAEK) is stained to verify its suitability for transplantation. Peculiar mortality lines possibly linked to hyperpressurization during the microkeratome cut can be seen in the paracentral region.
